# Effect of Aerobic Training on Glucose Control and Blood Pressure in T2DDM East African Males

**DOI:** 10.1155/2014/864897

**Published:** 2014-03-04

**Authors:** Huimin Yan, Antonio Prista, Sushant M. Ranadive, Albertino Damasceno, Paula Caupers, Jill A. Kanaley, Bo Fernhall

**Affiliations:** ^1^Exercise and Cardiovascular Research Laboratory, Department of Kinesiology and Community Health, University of Illinois at Urbana-Champaign, Champaign, IL 61801, USA; ^2^Faculty of Physical Education and Sports Sciences, Universidade Pedagógica, 1100 Maputo, Mozambique; ^3^Faculty of Medicine, Eduardo Mondlane University, 1100 Maputo, Mozambique; ^4^Department of Nutrition and Exercise Physiology, University of Missouri, Columbia, MO 65211, USA; ^5^Department of Kinesiology and Nutrition, University of Illinois at Chicago, Chicago, IL 60612, USA

## Abstract

*Background.* Exercise training intervention is underused in the management of type 2 diabetes mellitus in East Africa. *Methods.* 41 physically-active males with type 2 diabetes mellitus living in Mozambique were recruited and randomly assigned to 12 weeks of supervised exercise of low intensity exercise (LEX), vigorous intensity exercise (VEX), or to a control group (CON). Since there were no differences for any outcome variables between the exercise groups, VEX and LEX were combined into one exercise group (EX). *Results.* Age and baseline body weight were similar between EX and CON. Plasma glucose at 120 min following glucose load (Glu 120) was significantly reduced in the EX group after training (Glu 120 : 17.3 mmol/L to 15.0 mmol/L, *P* < 0.05), whereas Glu 120 remained unchanged in the CON (Glu 120 : 16.6 mmol/L to 18.7 mmol/L). After controlling for baseline blood pressure (BP), posttraining systolic BP and diastolic BP were lower in the EX group than in the CON group (EX: 129/77 mm Hg, CON: 152/83 mm Hg, *P* < 0.05). *Conclusion.* Adding exercise to already active African men with type 2 diabetes improved glucose control and BP levels without concomitant changes in weight.

## 1. Introduction

Diabetes is an increasing cause of morbidity and mortality in Africa. According to the World Health Organization (WHO), in 2000, there were 133,000 known individuals with type 2 diabetes in Mozambique and possibly an equal number of undiagnosed cases. Globally there is a rapid increase in the incidence of diabetes but developing countries have contributed substantially to the increased incidence [1], mainly as a consequence of urbanization [2]. It is projected that, by 2030, the number of people with diabetes will more than double in Mozambique.

Urbanization is associated with altered diet and decreased physical activity [2]. Although physical activity levels are declining in developing countries, they are still considerably higher than developed countries, especially in rural areas [3]. In Mozambique, many individuals walk more than one hour per day, as walking is a primary mode of transportation.

Exercise is widely prescribed in developed countries as a lifestyle intervention to control glucose and blood pressure (BP) in type 2 diabetic patients [4–7]. Because of their inherent daily physical activity in developing countries, exercise is seldom prescribed to patients with type 2 diabetes. Therefore, little is known if a structured exercise program added to an already active lifestyle would affect glucose control and BP in this population with type 2 diabetes. We hypothesized that a 12-week (wk) aerobic exercise program would improve glucose control and lower BP in already active type 2 diabetics living in Mozambique.

## 2. Methods

### 2.1. Subjects

Forty-one male subjects, between the ages of 40 and 70, diagnosed with type 2 diabetes for a minimum of 12 months were recruited from a local diabetes clinic in Maputo, Mozambique, and 41 subjects completed the study. All subjects gave written consent. This study was approved by the local Institutional Review Board. All subjects walked a minimum of one hour per day as part of their daily lifestyle and were free from any known disease other than type 2 diabetes and hypertension, including diagnosed cardiovascular disease.  Table 1 summarizes the medications used by the exercise intervention (EX) and control group (CON).

### 2.2. Measurements

Three resting systolic BP (SBP) and diastolic BP (DBP) measurements were obtained using an automated cuff (Dynamap, Waukesha, WI) following 10 minutes (min) of seated rest and with 2-minute intervals between each measurement. The mean of these measurements was used. Body weight was measured and body mass index (BMI/kg/m^2^) was calculated. Resting heart rate (HR) was recorded. Subcutaneous fat was measured with skinfold calipers taken at the chest, abdomen, and thigh. Fasting blood samples were collected at rest prior to a 75 g glucose load and at 120 min postingestion to determine glucose handling. Glycosylated haemoglobin (HbA1c) was analyzed from resting blood samples. Glucose and HbA1c were analyzed commercially by a clinical laboratory (LAC, Lda., Maputo, Mozambique). VO_2_ peak was evaluated from treadmill walking with metabolic measurements (MedGraphics, Minneapolis, MN) using a Balke walking protocol [8]. The test was terminated when subjects met three of the following five criteria: (i) a final rating of perceived exertion score of ≥17 on the Borg scale (scale 6–20), (ii) a respiratory exchange ratio >1.1, (iii) no change in HR with a change in workload, (iv) a “plateau” (increase of no >150 mL) in oxygen uptake with an increase in workload, and (v) volitional fatigue, defined as an inability to keep up with the treadmill speed.

### 2.3. Intervention

These subjects were randomly allocated to either low intensity (LEX, *n* = 22), vigorous intensity (VEX, *n* = 9) exercise intervention, or to the control group (CON, *n* = 10) for 12 weeks. The LEX group underwent supervised exercises at 50% VO_2_ peak for 45 min/session, 3–5 times/week, and the VEX group exercised under supervision at 75% VO_2_ peak for 45 min/session, 3 times/week.

### 2.4. Statistics

Statistical analyses were performed using the Statistical Package for Social Sciences (SPSS, version 16.0 for Windows). Baseline comparisons were made using independent *t*-tests. Since there were no differences for any outcome variables between the exercise groups, VEX and LEX data were combined into one exercise group (EX). Data were analyzed using a 2 × 2 (group (exercise versus controls) × pre/post-training) ANOVA with repeated measures. ANCOVA was used to compare postintervention BP while controlling for baseline BP variances. A Pearson *r* correlation was used to determine whether significant correlations existed between changes in HbA1c and SBP and HbA1c and DBP.

## 3. Results

**Figure 1:**
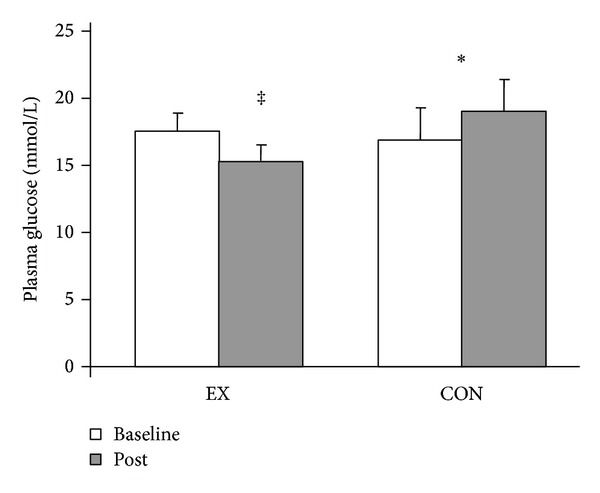
Plasma glucose at 120 min during oral glucose tolerance test at baseline and postintervention in EX and CON groups. *P < 0.05 interaction effect; ^‡^ *P* < 0.05 between EX and CON.

**Figure 2:**
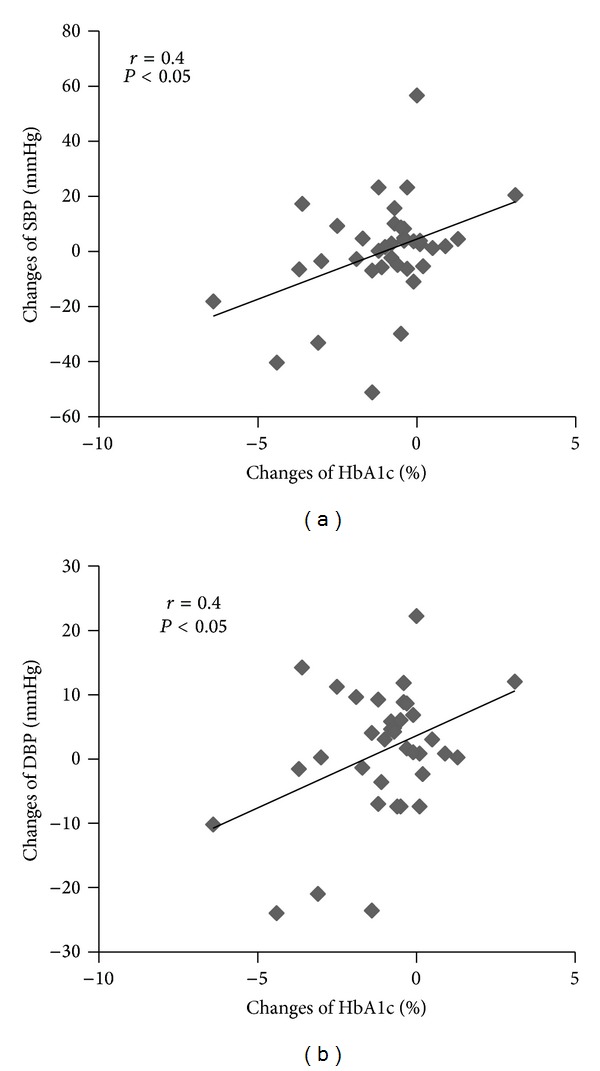
Changes of HbA1c was significantly correlated with (a) changes of SBP (*P* < 0.05) and (b) changes of DBP (*P* < 0.05) after intervention.

Subject characteristics and comparison between EX and CON at baseline and postintervention are shown in Table 2. There were no differences in age, height, and baseline weight between the EX and CON groups. SBP and DBP at the end of the intervention were significantly lower in the EX than CON group after covarying for the baseline values (*P* < 0.05). HbA1c was significantly reduced in both groups combined, but there was no significant interaction effect. In response to the glucose load, the EX group had a significantly lower plasma glucose level than CON group (*P* < 0.05) following the exercise training (Figure 1). Skinfold measurement revealed a reduction in subcutaneous chest and thigh fat in the EX group only after training. The change of HbA1c (−1.0 ± 0.3%) over the 12 weeks was significantly correlated with the change of SBP (0.3 ± 2.7 mm Hg, *r* = 0.4, *P* < 0.05) and DBP (−1.4 ± 1.5 mm Hg, *r* = 0.4, *P* < 0.05) (Figure 2).

## 4. Discussion

Individuals with type 2 diabetes are encouraged to be physically active, yet it is not known whether adding exercise to physically active individuals with type 2 diabetes would improve glucose control. This study has demonstrated that adding a structured exercise intervention to already physically active East African males with type 2 diabetes improved glucose control over 12 weeks. In addition, improvement in resting BP and exercise capacity were also observed in the EX group and these improvements occurred without weight loss.

In the current study, the intensity and duration of the exercise training program met the recommendations made by public health guidelines for type 2 diabetes [9]. Despite unchanged VO_2_ peak of EX over the 12 wks, treadmill time during a VO_2_ max test was increased after training, indicating improvement in work capacity. Considering that the participants already walked a minimum of 1 hr per day, this finding indicates that addition of more intense exercise to already physically active individuals can improve work capacity, without significant changes in VO_2_ max.

Lowering BP in diabetics is clinically beneficial. Hypertension, especially high SBP [10], is associated with diabetic nephropathy, retinopathy, neuropathy, and cardiovascular disease [11–13]. In the UK Prospective Diabetes Study (UKPDS), significantly fewer diabetic micro- and macrovascular complications and diabetic-related deaths occurred in the group with a mean BP of 144/82 mm Hg compared to the group with a mean BP of 154/87 mm Hg [14]. The reduction in resting BP in the EX group observed in the present study was in accordance with previous findings, in which 12 wks of aerobic training decreased BP in Africans with hypertension [15]. In our study, the exercise program reduced the mean SBP by 2 mm Hg, whereas the mean SBP in CON group increased 7 mm Hg. It has been estimated that a 2-mm Hg reduction of SBP results in a 6% reduction in stroke mortality and a 4% reduction in mortality attributable to coronary heart disease [16], thus, even the small reduction in BP observed in our study is clinically significant. Mean DBP was decreased in the EX group by 3 mm Hg while increased in the CON group by 3 mm Hg. Small decrements in DBP of 3 mm Hg reduces the risk of stroke 25% and risk of coronary artery disease by 9% [17]. Although our BP findings may have been influenced by the baseline BP, the BP was not reduced and actually increased in the CON group. We further confirmed this finding by covarying for baseline SBP in the statistical analysis and finding significantly lower postintervention SBP in the EX group. Thus, it is unlikely our results can be explained by “regression towards to the mean”. The ~2 mm Hg reduction in SBP and ~3 mm Hg reduction in DBP are similar to the expected reduction from exercise training reported in a recent meta-analysis [16], suggesting that our EX group exhibited a standard reduction in BP in response to exercise training.

The decrease in HbA1c by 1.1% in EX is clinically significant, since a reduction in HbA1c by 0.6% was shown to reduce the risk of microvascular complications by 25% [18]. This finding is in agreement with Ronnemaa et al., who reported four months of physical exercise decreased HbA1c from 9.6 to 8.6% [19]. A recent review of the response of HbA1c levels to exercise training found a modest response (0.5–1.0% reduction) or no response to training interventions [20]. The decrease in HbA1c by 0.7% in CON group is also clinically significant. The finding is in agreement with that of van Rooijen et al. [21], who reported reduced HbA1c in a relaxation group to the same extent as the exercise group in black female diabetics.

Exercise training had little impact on resting plasma glucose [22], but response to the glucose challenge was attenuated, while the CON group demonstrated no change, suggesting that additional exercise in physically active individuals can enhance glucose control. The disparity of improvements in the 2 hr glucose level while there is no change in fasting glucose responses is in agreement with Krotkiewski et al. [5]. In their study, although fasting plasma glucose was unaltered, exercise training improved type 2 diabetic patients' performance during glucose tolerance tests. This evidence suggests that exercise training may provide a unique impact on postprandial glucose disposal, independent of weight loss or pharmacological agents used. Thus, our data show oral glucose tolerance was improved with a formal exercise training program in already active individuals with type 2 diabetes. Further research is needed to identify the mechanisms mediating the differential responses.

Both EX and CON exhibited small but significant increases in body mass and BMI compared to the baseline. The increase in body mass in the EX group implies that either the patients increased their daily energy intake or they diminished their habitual physical activity level outside the scheduled training, or their muscle mass was increased, or combined. Due to the difficulties of translating exercise history questionnaires into different lingual dialects, we failed to collect valid daily activity data outside the exercise intervention. However, local people use walking as their major means of transportation, and based on participant interviews, we are confident that they walked for at least 1 hour every day at baseline.

Physical activity may decrease total fat mass and visceral fat without weight loss in obese type 2 diabetics [4, 6]. Excess fat, especially visceral fat, is closely associated with insulin resistance and the pathology of type 2 diabetes [23]. Although data from skinfold measurements indicated small changes in fat distributions in EX, waist circumference was unchanged. Thus, it is unlikely that abdominal fat changed in our study. It is also important to note that the diabetic population in the current study was much leaner than diabetics typically studied in western society. Based on average BMI of 27 and waist-hip ratio less than 1.0, the subjects in our study were not obese according to WHO standards. Although weight loss is a desirable strategy of treating diabetes in developed counties, in developing countries where physical activity is prevalent in their lifestyle, males with type 2 diabetes benefit from additional exercise training even without weight loss. This supports previous data demonstrating that exercise alone can improve glucose metabolism in type 2 diabetics [6]. Exercise training increases glucose disposal and decreases muscle insulin resistance through a number of mechanisms that may not necessarily be associated with weight loss. Insulin increases glucose transport in muscle by translocating GLUT 4 glucose transporting protein from intracellular stores to sarcolemma [24]. Training-induced changes by muscle contraction include increases in muscle GLUT 4 concentration, GLUT 4 mRNA [7], and glycogen synthase activity and changes in muscle composition, favoring increased glucose disposal [25]. A close correlation between glycogen synthase fractional activity and blood flow suggests that they are causally related in promoting glucose disposal [26]. Activation of the sympathetic nervous system and hypertension are associated with insulin resistance [27]. We have found a relationship between the changes of HbA1c and the changes in BP in our study. Because exercise training improves insulin sensitivity and glucose metabolism [28], this may be an important mechanism in mediating reductions in sympathetic outflow and BP. This would be consistent with previous data in hypertensive subjects demonstrating a close association between the reduction in resting BP and plasma norepinephrine and improved insulin sensitivity after exercise training [29]. This may at least partially explain the favorable BP and glucose metabolism changes we observed in our study.

*Limitations*. Data on plasma insulin was not available because of a malfunction in the lab. We were also unable to collect reliable data on baseline and postprogram physical activity. Our findings may not apply to women, or to other populations in Africa with lifestyles different from those in Mozambique. Despite these limitations, the study provides insight on the effect of exercise training on individuals with type 2 diabetes in East Africa.

## 5. Conclusions

Adding exercise to already active African men with type 2 diabetes improved glucose control and BP without concomitant weight loss; thus additional exercise should be encouraged in those individuals who appear to have adequate physical activity levels. Exercise training can be viewed as beneficial on its own, independent of weight loss.

## Figures and Tables

**Table 1:** A summary of medications.

	EX	CON	Classification
Nifedipine	0 (0)	1 (10)	antihypertensive
Amiloride	8 (20)	3 (30)	antihypertensive
Hydrochlorothiazide	16 (39)	6 (60)	antihypertensive
Methyldopa	4 (10)	0 (0)	antihypertensive
Enalapril	12 (29)	1 (10)	antihypertensive
Chlorthalidone	3 (7)	0 (0)	antihypertensive
Metformin	13 (32)	3 (30)	antidiabetic
Glyburide	25 (61)	6 (60)	antidiabetic
Atenolol	1 (2)	1 (10)	antihypertensive

Numbers are expressed as count (percentage within this group).

Many subjects were on multiple medications.

**Table 2:** Comparison between EX and CON at baseline and after intervention.

	EX		CON	
	Baseline	Post	Baseline	Post
Age	53 ± 2	—	55 ± 3	—
Height	170.0 ± 1.2	—	170.3 ± 1.3	—
Weight (kg)^#^	78.8 ± 2.4	79.7 ± 2.4	78.4 ± 4.3	79.7 ± 4.3
BMI^#^	27.2 ± 0.7	27.4 ± 0.7	27.0 ± 1.3	27.5 ± 1.3
Waist circumference (cm)	95.8 ± 2.2	94.2 ± 1.9	95.1 ± 3.7	95.0 ± 2.2
Hip circumference (cm)	99.7 ± 1.5	98.7 ± 1.5	98.9 ± 2.6	98.2 ± 2.6
SBP (mmHg)	131 ± 3^‡^	129 ± 4^§^	145 ± 6	152 ± 6
DBP (mmHg)	80 ± 1^‡^	77 ± 2^§^	80 ± 3	83 ± 3
Plasma glucose (mmol/L)	10.3 ± 0.9	9.6 ± 0.7	9.0 ± 1.5	11.1 ± 1.3
HbA1c (%)^#^	8.8 ± 0.5	7.7 ± 0.4	8.4 ± 0.9	7.7 ± 0.8
Time on treadmill (min)*	10.7 ± 0.7	12.4 ± 0.8^†^	9.8 ± 1.7	9.6 ± 1.8
VO_2_ max (mL/kg/min)	23.1 ± 1.0	25.0 ± 1.3	21.6 ± 2.5	22.5 ± 3.0
Chest skinfold (cm)^#^	19.3 ± 1.4	14.8 ± 1.0^†^	21.8 ± 3.0	16.2 ± 2.1
Abdominal skinfold (cm)	22.7 ± 1.2	22.8 ± 1.5	21.3 ± 2.6	23.0 ± 3.2
Thigh skinfold (cm)*	16.3 ± 1.3	14.1 ± 1.5^†^	15.8 ± 2.7	16.2 ± 3.0
Total skinfold (cm)^#^	59.0 ± 3.2	52.4 ± 3.4^†^	58.9 ± 3.4	55.4 ± 7.0

Note: values are mean ± SEM;

VO_2_ max: maximal aerobic capacity;

BMI: body mass index;

*P < 0.05 interaction effect; ^#^ *P* < 0.05 time effect; ^§^ *P* < 0.05 between EX and CON after covarying for baseline; ^‡^ *P* < 0.05 between EX and CON; ^†^ *P* < 0.05 between baseline and post in EX.
